# T cell specific adaptor protein (TSAd) promotes interaction of Nck with Lck and SLP-76 in T cells

**DOI:** 10.1186/s12964-015-0109-7

**Published:** 2015-07-11

**Authors:** Cecilie Dahl Hem, Vibeke Sundvold-Gjerstad, Stine Granum, Lise Koll, Greger Abrahamsen, Laszlo Buday, Anne Spurkland

**Affiliations:** Department of Molecular Medicine, Institute of Basic Medical Sciences, University of Oslo, Oslo, 0317 Norway; Institute of Enzymology, Research Centre for Natural Sciences, Hungarian Academy of Sciences, Budapest, 1117 Hungary; Institute of Basal Medical Sciences, University of Oslo, PB 1105, Blindern, Oslo 0317 Norway

**Keywords:** TSAd, T cell specific adaptor protein, Nck, Lck, SH2 domain, adaptor protein, SH2D2A, SLP-76

## Abstract

**Background:**

The Lck and Src binding adaptor protein TSAd (T cell specific adaptor) regulates actin polymerization in T cells and endothelial cells. The molecular details as to how TSAd regulates this process remain to be elucidated.

**Results:**

To identify novel interaction partners for TSAd, we used a scoring matrix-assisted ligand algorithm (SMALI), and found that the Src homology 2 (SH2) domain of the actin regulator Non-catalytic region of tyrosine kinase adaptor protein (Nck) potentially binds to TSAd phosphorylated on Tyr^280^ (pTyr^280^) and pTyr^305^. These predictions were confirmed by peptide array analysis, showing direct binding of recombinant Nck SH2 to both pTyr^280^ and pTyr^305^ on TSAd. In addition, the SH3 domains of Nck interacted with the proline rich region (PRR) of TSAd. Pull-down and immunoprecipitation experiments further confirmed the Nck-TSAd interactions through Nck SH2 and SH3 domains. In line with this Nck and TSAd co-localized in Jurkat cells as assessed by confocal microscopy and imaging flow cytometry. Co-immunoprecipitation experiments in Jurkat TAg cells lacking TSAd revealed that TSAd promotes interaction of Nck with Lck and SLP-76, but not Vav1. TSAd expressing Jurkat cells contained more polymerized actin, an effect dependent on TSAd exon 7, which includes interactions sites for both Nck and Lck.

**Conclusions:**

TSAd binds to and co-localizes with Nck. Expression of TSAd increases both Nck-Lck and Nck-SLP-76 interaction in T cells. Recruitment of Lck and SLP-76 to Nck by TSAd could be one mechanism by which TSAd promotes actin polymerization in activated T cells.

## Background

Regulation of actin dynamics is important for several aspects of T cell function, including differentiation, migration through tissues and proliferation. T cell activation initiates multiple molecular events including activation of protein tyrosine kinases, the formation of protein signaling complexes, and cytoskeletal actin reorganization leading to establishment of the immunological synapse (IS) at the T cell-antigen presenting cell (APC) interface [1, 2]. Briefly, following T cell receptor (TCR) ligation the tyrosine kinases Lck and Zap-70 become activated, leading to the formation of a signaling complex containing the adaptor proteins linker for activation of T cells (LAT), SH2 domain containing leukocyte protein of 76 kDa (SLP-76) and non-catalytic region of tyrosine kinase adaptor protein (Nck) [2].

The molecular aspects concerning actin dynamics in T cells are complex, and many questions remain to be solved. One of the more recently identified players of actin reorganization is the T cell specific adaptor protein (TSAd) [3] encoded by the *SH2D2A* gene. TSAd interacts with and modulates the activity of the Src family protein tyrosine kinase Lck [4, 5] as well as Src itself [6]. TSAd has been found to control actin polymerization events in T cells and endothelial cells. More specifically, in response to VEGF-A stimulation, TSAd is required for stress fiber formation and migration of endothelial cells [7]. Moreover, we have also shown that TSAd regulates CXCL12-induced migration and actin cytoskeletal rearrangements in T cells by promoting Lck dependent tyrosine phosphorylation of IL2-inducible T-cell kinase (Itk) [8].

To better understand the function of TSAd, we used an algorithm for identification of SH2 domain-ligand pairs (SMALI) to identify possible binding partners for the TSAd phosphotyrosines. SMALI pointed to a possible interaction between TSAd and the adaptor Nck. Nck is known to regulate the actin cytoskeleton. It consists of one C-terminal Src homology 2 (SH2) domain and three N-terminal SH3 domains which allows for multiple protein-protein interactions. More than 60 interaction partners for Nck have been identified [9, 10]. Nck interacts constitutively with the guanine nucleotide exchange factor Vav1 [11]. Upon TCR-triggering, Nck and Vav1 interacts with SLP-76, leading to the activation of the actin rearrangement at the T-cell APC interface. Thus, Nck is a key adaptor in T cell activation-dependent actin filament formation through its interactions with components of the TCR/CD3 complex and cytoskeletal regulators including Vav1 and SLP-76 [9, 12–14]. Nck plays a universal role in regulation of the signaling networks critical for organizing the actin cytoskeleton; including formation of the IS following TCR engagement, cell proliferation and cell migration [9, 15, 16].

Here we explored the possible interaction between TSAd and Nck using intact and mutated TSAd and Nck constructs. We found that the Nck SH2 domain binds to both TSAd pTyr^280^ and TSAd pTyr^305^, with pTyr^280^ as the preferred binding site. Additionally, two of the three Nck SH3 domains were found to interact with the PRR on TSAd, presumably in a cooperative manner. Our data indicate the existence of a direct interaction between of Nck and TSAd. When TSAd is co-expressed, interaction of Nck with Lck is increased. Moreover, TSAd also enables Nck to interact with SLP-76, an interaction previously shown to be important for actin polymerization and rearrangement [17]. TSAd promoted actin polymerization in Jurkat cells, and this was dependent on TSAd exon 7 encoding interaction sites for both Nck and Lck. Thus, the Nck-TSAd interaction may represent an additional link whereby TSAd contributes to the regulation of the actin cytoskeleton in T cells.

## Results

### The Nck SH2 domain interacts with TSAd-pTyr^280^ and -pTyr^305^

**Fig. 1:**
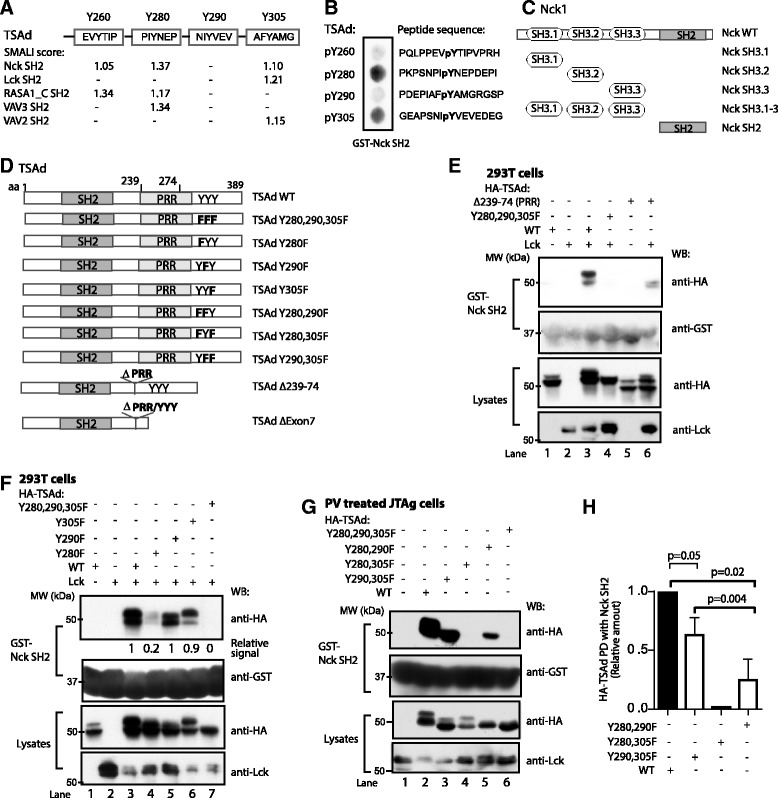
TSAd-pTyr^280^ and -pTyr^305^ interact with the Nck SH2 domain. **a** SH2 domain interaction partners to TSAd phosphotyrosines predicted by SMALI. The five best candidates are listed. Scores > 1 indicate a potential interaction.”-“indicates a SMALI score < 1. **b** Peptide spot array spotted with indicated TSAd phosphopeptides probed with GST-Nck SH2 and developed with anti-GST antibody. SH2: Src homology 2 domain, Y: tyrosine phosphorylation site. **c** and **d** Overview of the Nck (**c**) and TSAd (**d**) Constructs used. WT: wild type, SH3: Src homology 3 domain, PRR: proline rich region, Y: tyrosine phosphorylation site and F: phenylalanine. **e-g** Proteins pulled down by GST-Nck SH2 were resolved on SDS-PAGE, and immunoblotted with the indicated antibodies. **e** GST-Nck SH2 pull-down from 293T cells transiently transfected with Lck and the indicated HA-tagged TSAd cDNA constructs. Lck immunblot of the GST-Nck SH2 pulldown was negative (data not shown). **f** As in (e), including TSAd encoding single Y → F mutations. Relative TSAd binding to GST-Nck SH2 domain was analysed by ImageJ. Amount of pulled down HA-TSAd WT was set to 1. **g** GST-Nck SH2 pull-down from pervanadate treated JTAg cells transiently expressing indicated TSAd Y → F double mutations. Control GST pull-downs were negative (data not shown). Data shown are representative of at least three experiments (triple Y → F TSAd mutant and double mutants) or two experiments (Y → F single mutants). **h** Graph shows relative amounts of HA-TSAd that were pulled down (PD) with GST-Nck SH2 as in (g) measured by ImageJ. Amount of pulled down HA-TSAd WT was set to 1. Mean values ± SD of three independent experiments (2-tailed paired *t*-test)

TSAd possesses several protein interaction motifs, including an N-terminally located SH2 domain, and a C-terminal part consisting of a PRR and several tyrosine phosphorylation sites. TSAd is tyrosine phosphorylated in non-stimulated Jurkat cells [4, 18] and in peripheral blood mononuclear cells [3] while increased amount of tyrosine phosphorylated TSAd may be seen upon TCR stimulation [18]. To identify novel SH2 domain containing binding partners for TSAd, we performed an *in silico* scan using the SMALI algorithm [19, 20]. A relative SMALI score >1.0, strongly indicates potential binding between an SH2 domain and a phosphotyrosine containing ligand. SMALI identified the Nck SH2 domain as a possible interaction partner for TSAd pTyr^260^, pTyr^280^ and pTyr^305^, (relative SMALI scores: 1.05, 1.37 and 1.10 respectively) (Fig. 1a). In comparison, the Lck-SH2 domain already known to bind to the three C-terminal TSAd phosphotyrosines [5], displayed a SMALI score of 1.21 for TSAd-pTyr^305^. To test the SMALI predictions, we carried out a peptide array analysis of 15-mer TSAd phosphotyrosine peptides spotted to a nitrocellulose membrane. Recombinant GST-Nck SH2 protein showed direct binding to both TSAd pTyr^280^ and pTyr^305^ while the TSAd pTyr^260^ phosphopeptide only revealed a weak signal (Fig. 1b). In accordance with the SMALI predictions, the pTyr^280^ interaction displayed the strongest signal (Fig. 1b).

Interaction between Nck and TSAd was then assessed by *in vitro* pull-down interaction analysis in lysates from 293T cells transiently expressing TSAd as well as the kinase Lck to ensure phosphorylation of TSAd [4, 18]. The constructs used for transfections are depicted in Fig. 1c and d. TSAd exists in different phosphorylation forms, with different migration in SDS-PAGE [5]. In the absence of Lck, the lower band predominates (Fig. 1e, lane 1). Nck SH2 protein pulled down TSAd or TSAd lacking its PRR from 293T cells only when co-expressed with Lck (Fig. 1e, lane 3 and 6). In contrast TSAd lacking the three penultimate tyrosines known to be phosphorylated by Lck [5], failed to interact (Fig. 1e, lane 4). Moreover, Nck SH2 did not interact with Lck in this experiment (data not shown). We previously showed that removal of the TSAd PRR is associated with reduced phosphorylation of TSAd [5]. In line with this observation, the absence of TSAd PRR results in reduced interaction between the GST-Nck SH2 domain and TSAd (Fig. 1e, lane 6) compared to TSAd WT.

Further analysis revealed that substantially less of the TSAd Tyr^280^ → Phe^280^ (Y280F) mutant was pulled down with Nck SH2, while Nck SH2 could still pull down the TSAd Y305F protein (Fig. 1f). Elimination of Tyr^290^ did not affect TSAd’s binding to Nck SH2 in 293T cells (Fig. 1f), as predicted by SMALI and shown in the peptide array analysis (Fig. 1b). GST alone did not bind to TSAd (data not shown). The results presented in Fig. 1f revealed that single mutations of TSAd Tyr^280^, Tyr^290^ and Tyr^305^ did not completely abolish binding between TSAd and the Nck SH2 domain. We thus proceeded to analyze double Y to F mutants of TSAd for interaction with Nck SH2 using pull-down experiments in Jurkat TAg (JTAg) cells transiently expressing HA-tagged TSAd (Fig. 1g). To ensure maximal tyrosine phosphorylation of TSAd we chose to treat the TSAd transfected JTAg cells with the strong tyrosine phosphatase inhibitor pervanadate (PV) prior to cell lysis and pull down with Nck SH2. Mutation of both Tyr^280^ and Tyr^305^ on TSAd (Y280, 305F) resulted in disrupted interaction between TSAd and GST-Nck SH2 (Fig. 1g, lane 4), while TSAd molecules with either Tyr^280^ or Tyr^305^ intact retained ability to interact with Nck SH2. This pattern of Nck SH2 interaction could reflect differences in tyrosine phosphorylation as TSAd pTyr^280^ is reported twice as frequently in the phophosite.org database as TSAd pTyr^305^ (Gopalakrishna et al. *submitted*). However, both TSAd Tyr^280^ and Tyr^305^ are phosphorylated by Lck to a similar extent in vitro [21], thus the difference in amount of TSAd mutants pulled down could also reflect differences in binding affinity, as indicated by SMALI (Fig. 1b). Taken together, these results confirm the SMALI predicted interaction between the Nck SH2 domain and TSAd pTyr^280^ and pTyr^305^, with pTyr^280^ as the preferred binding site.

### The proline rich region of TSAd interacts with the SH3 domains of Nck

**Fig. 2:**
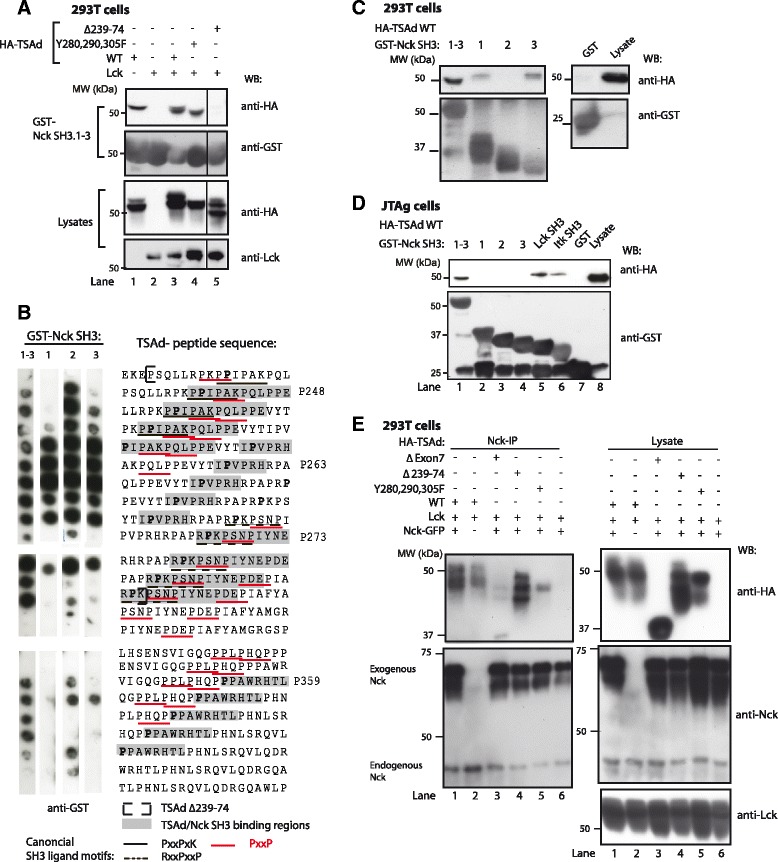
The proline rich region (PRR) of TSAd interacts with the Nck SH3 domains. **a** GST-Nck SH3 pull-downs from 293T cells transiently expressing the indicated HA-tagged TSAd constructs. Proteins were resolved on SDS-PAGE, and immunoblotted with the indicated antibodies. Control GST pull-downs were negative (data not shown). Lck immunblot of the GST-Nck SH3 pulldown was negative (data not shown). Results shown are from the same experiment as shown in Fig. 1e and are representative of at least three experiments. **b** Peptide spot array spotted with proline containing TSAd peptides probed with GST-Nck SH3 peptides and developed using anti-GST antibody. Nck SH3 domain interaction sequences on TSAd are boxed in grey, the TSAd region aa 239–274 is outlined and canonical SH3 ligand motifs and the core PXXP motifs are underlined with black or red lines respectively. **c** Pull-down with single GST-Nck SH3 domains and control GST performed as in (a). Results are one representative of three independent experiments. **d** Pull-down of GST-Nck SH3 domains in JTAg cells transiently expressing HA-TSAd WT. Positive controls: Itk SH3 and Lck SH3. Negative control: GST. Blots were immunoblotted with the indicated antibodies. Data is one representative of two experiments. **e** Nck co-immunoprecipitation of TSAd expressed in 293T cells. Cells were transiently transfected with Nck-GFP, Lck and the indicated HA-tagged TSAd constructs. Cell lysates and anti-Nck IP were separated by SDS-PAGE and immunoblotted with indicated antibodies. Data are representative of three independent experiments

In addition to one SH2 domain, Nck contains three N-terminally located SH3 domains (Fig. 1c). PRRs provide possible interaction sites for SH3 domains, and we have previously shown that TSAd aa 239–274 harbors binding sites for Lck and Itk SH3 domains [5, 8]. We thus examined whether the TSAd PRR could bind to any of the three Nck SH3 domains. A Scansite search [22] at low stringency identified the Nck SH3.2 domain as a potential binding partner for TSAd prolines aa 245 (EPSQLLR**P**KPPIPAK) and aa 353 (SVIGQGP**P**LPHQPPP). Pull-down experiments in 293T cells (similar to Fig. 1e) expressing Lck and various TSAd constructs revealed binding of Nck SH3.1-3 domains to TSAd (Fig. 2a, lane 1). Neither presence of Lck (Fig. 2a, lane 1 and 3) nor mutation of the three penultimate tyrosines (Y280,290,305 F) of TSAd (Fig. 2a, lane 4) affected the interaction with Nck SH3.1-3 while the PRR (∆239-274) was required for binding to GST-Nck SH3.1-3 (Fig. 2a, lane 5). Lck has been reported to interact with Nck-SH3 domains [23]. However, Lck was not found to interact with Nck-SH3.1-3 in this experiment (data not shown). These results indicate binding of the Nck SH3 domains to the PRR of TSAd.

We then studied the TSAd interaction to individual Nck SH3 domains in more detail. Peptide spot arrays of 20-mer peptides representing the TSAd PRR showed direct binding of full length GST-Nck SH3.1-3 domains and all three GST-Nck SH3 single domains to TSAd peptides (Fig. 2b). Several classical SH3-ligand motifs are found within the TSAd PRR peptides that bind to the Nck SH3 domains, including the minimal consensus sequence PxxP, and the canonical K/RxxPxxP and PxxPxK/R motifs. Binding of Nck SH3 domains to TSAd prolines were found to occur primarily within the TSAd PRR aa 239–274. This observation is in agreement with results from pull-down data (Fig. 2a), showing abolished Nck SH3 domain interaction with TSAd Δ239-274. In comparison, although the C-terminal TSAd sequence aa 359-366 (P359) displayed some reactivity with the Nck-SH3 domains, this part of TSAd probably does not represent important Nck SH3 interaction sites. There are no classical SH3 domain binding motifs within this region, and pull-down experiments showed no binding of Nck SH3 domains to TSAd ∆239-274 (Fig. 2a) where aa 359–366 are present.

To confirm interaction of Nck SH3 domains with TSAd, we performed pull-down assays from 293T cell expressing TSAd using single GST-Nck SH3 domain constructs. GST-Nck SH3.1 and SH3.3 both interacted with TSAd (Fig. 2c). The Nck SH3 interaction with TSAd was confirmed in JTAg cells transfected with plasmids encoding intact HA-tagged TSAd. All three Nck SH3 domains together (1–3) pulled down similar amounts of TSAd as Lck-SH3 (Fig. 2d, compare lane 1 and 5), while single Nck SH3 domains did not pull-down TSAd (Fig. 2d, lane 2–4). The discrepancy between Nck SH3 binding in 293T and JTAg cells regarding the single Nck SH3 domains may be due to other competing Nck-SH3 ligands present in JTAg cells. The different migration of HA-TSAd that interacted with Nck SH3.1-3 versus single Nck, Lck or Itk SH3 domains could be due to in-gel interference between the GST-fusion proteins and TSAd, either due to the molecular size of the GST protein, or the number of binding sites for TSAd. Taken together, these results show that Nck may interact with TSAd also through its SH3 domains, and that the three SH3 domains interact cooperatively with TSAd.

### Both TSAd phosphotyrosines and PRR contribute to TSAd-Nck interaction

The interaction between TSAd and Nck was further analyzed in Nck immunoprecipitates (IPs) from 293T cells expressing Lck together with wild type (WT) or mutated HA-tagged TSAd molecules and GFP-tagged Nck molecules. TSAd WT co-precipitated with Nck (Fig. 2e, lane 1 and 2). Exon7 encodes most of the PRR and the three C-terminal TSAd tyrosines (Fig. 1d), which includes the Nck interaction sites identified in Fig. 1e-g and Fig. 2a-c. TSAd encoded by cDNA lacking exon7 did not co-immunoprecipitate with Nck (Fig. 2e, lane 3). Removal of either the Nck SH2 or SH3 domain interaction sites on TSAd (∆239-274 or Y280,290,305F), did not fully abolish TSAd-Nck interaction (Fig. 2e, lane 4 and 5). Collectively, these results show that both the phosphotyrosines and the PRR of TSAd may be engaged in Nck binding.

### TSAd and Nck co-localize

**Fig. 3:**
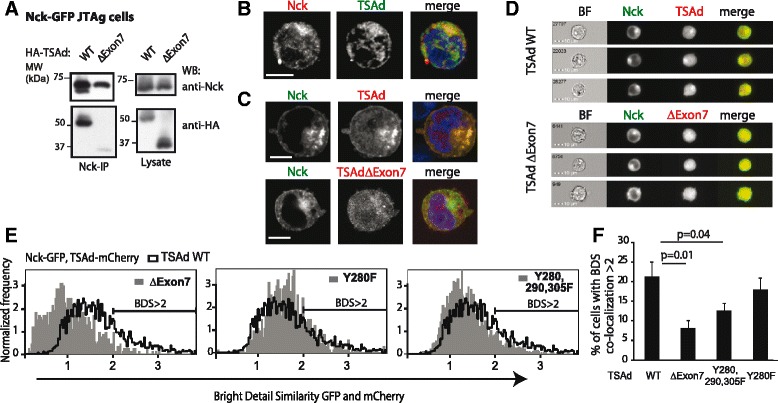
Co-localization of TSAd and Nck in T cells. **a** Nck co-immunoprecipitation of TSAd expressed in JTAg cells. Immunoblot of Nck-IP and lysates from JTAg cells transfected with Nck-GFP and HA-TSAd constructs. One representative of three experiments is shown. **b** Confocal microscopy images (60× magnification) of one CD3/28 activated human CD3+ T cell, stained with anti-Nck (red), anti-TSAd (green) and Hoechst 33342 (blue nuclear staining in the merged image). Scale bar = 5 μm. Images are representative of two independent experiments. **c** Confocal microscopy images (100× magnification) of transfected, fixed and permeabilized JTAg cells showing Nck-GFP (green), TSAd-mCherry (red) and merged images with nuclei staining Hoechst 33342 (blue). Images are representative of three independent experiments. Scale bar = 5 μm. **d** and **e** Co-localization of Nck-GFP and the indicated TSAd-mCherry molecules analyzed with the ImageStream cytometer, gated on double positive GFP and mCherry cells. **d** Gallery of representative IFC images. **e** Co-localization analyzed with the “Bright Detail Similarity” (BDS) algorithm in the IDEAS software. The BDS score is the log transformed Pearson's correlation coefficient of the localized bright spots with a radius of 3 pixels or less within the masked area in the two input images. Overlay of BDS histograms comparing co-localization of various TSAd molecules to Nck are shown. Data are representative of four independent experiments. **f** Percentage of cells that shows high degree of co-localization (BDS score >2) in (e) for Nck-GFP with each of the TSAd constructs. Graph shows mean ± standard deviation from four experiments. P-values are indicated (2-tailed paired *t*-test)

To further assess whether TSAd and Nck interact in vivo, we initially performed co-immunoprecipitation experiments from transiently transfected JTAg cells expressing exogenous TSAd and Nck. TSAd and Nck were found to co-immunoprecipitate in these cells (Fig. 3a). We then used confocal microscopy to examine the localization of endogenous Nck and TSAd in CD3/CD28 activated human CD3+ T cells (Fig. 3b), as well as in JTAg cells transiently expressing Nck-GFP and TSAd-mCherry (Fig. 3c). This analysis indicated that TSAd and Nck co-localized in both human CD3+ T cells and in transfected JTAg cells. TSAd lacking both the Nck SH2 and SH3 interaction sites (TSAd ∆Exon7), did not co-immunoprecipitate nor co-localize with Nck (Fig. 3a and c). A faint band was observed for the TSAd ∆Exon7 co-immunoprecipitate with Nck (Fig. 3a). This band is not specific since it was similar to background (without the Nck antibody, data not shown).

Co-localization between Nck and intact or mutated TSAd molecules in JTAg cells was then quantified by imaging flow cytometry (IFC) (Fig. 3d) using the bright detail similarity feature of the IDEAS software (Fig. 3e and f). Compared to TSAd WT, mutation of the Nck SH2 interaction site TSAd Tyr^280^, did not significantly affect Nck co-localization (Fig. 3f). However, when expressing TSAd mutated for all three C-terminal TSAd-tyrosines (i.e. Y280,290,305F), co-localization with Nck was significantly reduced. Again, absence of both the PRR and the pTyr sites on TSAd (∆Exon7) resulted in disruption of Nck and TSAd co-localization (Fig. 3f). Taken together, these results support the notion that TSAd and Nck interact *in vivo*, and that both Nck SH2 and SH3 domains contribute to this interaction.

### TSAd promotes interaction between Nck and Lck

**Fig. 4:**
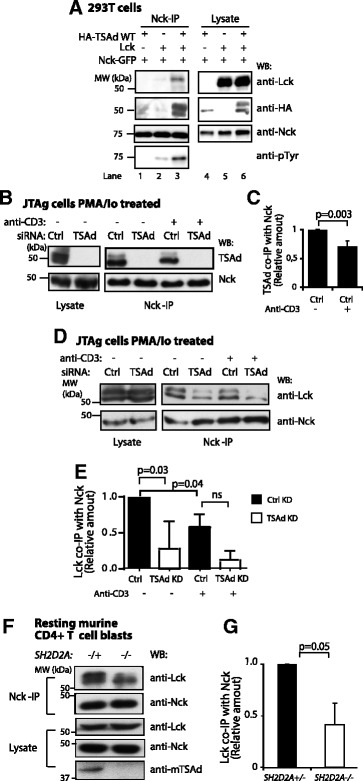
TSAd promotes Nck and Lck interaction. **a** Nck co-immunoprecipitation of TSAd and Lck protein expressed in 293T cells. 293T cells transfected with cDNA encoding Nck-GFP, HA-tagged TSAd and Lck were subjected to Nck-IP, and immunoblotted with the indicated antibodies. Data is one representative of three experiments. **b** and **c** Endogenous TSAd co-precipitate with Nck. Immunoblot of Nck-IP from JTAg cell lysates with TSAd expression (siRNA Control (Ctrl)) or TSAd knock down (siRNA TSAd (TSAd)). Cells were PMA/Io treated (20 h), and subsequently stimulated with anti-CD3 (1 min) or left unstimulated. Lysates were immunoblotted with the indicated antibodies. Results are one representative of three independent experiments. **c** Graph shows relative amounts of TSAd co-precipitated with Nck-IP as shown in (b) measured by densitometry (ImageJ). Mean values ± SD of three independent experiments (2-tailed paired t-test). **d** and **e** Co-precipitation of Lck with Nck. Cells were treated as described in (b). Amount Lck co-precipitated with Nck in unstimulated TSAd expressing cells (siRNA Control) was set to 1. KD: knock down. Mean values ± SD of three independent experiments (2-tailed paired *t*-test). ns: not statistically significant. **f** Nck-IP and lysates from resting CD4+ murine *SH2D2A* ^*−/+*^ or *SH2D2A* ^*−/−*^ blast T cells. Results shown are one representative out of three independent experiments. **g** Graph shows relative amount of Lck co-precipitated with Nck IP as shown in (f) as measured by ImageJ. Amount of Lck co-precipitated with Nck in *SH2D2A* ^*−/+*^ CD4+ T cells was set to 1. Mean values ± SD of three experiments. P-values are indicated (2-tailed paired *t*-test)

We and others have previously shown that the Src kinase Lck interacts with TSAd [4, 5, 18, 21, 24]. Additionally, Nck has been reported to bind to a proline motif in the unique domain of Lck [23]. We here examined whether TSAd may also serve as a molecular scaffold bringing Lck into the vicinity of Nck. We initially used 293T cells as these cells lack expression of T-cell specific proteins and thus provide a “pure” system to investigate T cell protein interactions. Cells were transiently transfected with Nck, Lck and/or TSAd cDNA constructs, and subjected to Nck-IP (Fig. 4a). No interaction of Nck with Lck was observed (Fig. 4a, lane 2). However, in cells co-expressing TSAd, Lck co-immunoprecipitated with Nck (Fig. 4a, lane 3). Moreover, while Nck was tyrosine phosphorylated in the presence but not in the absence of Lck, the level of Nck tyrosine phosphorylation was strongly increased in the presence of TSAd (Fig. 4a, pTyr-blot, compare lane 2 and 3).

To assess whether TSAd promotes Nck-Lck interaction also in T cells, we performed Nck co-immunoprecipitation in lysates from control JTAg cells or JTAg cells where endogenous TSAd expression had been suppressed by siRNA. Briefly, cells were stimulated with PMA and ionomycin over night to increase the amount of endogenous TSAd, washed, and restimulated with anti-CD3 antibodies. Nck co-precipitated with TSAd in these cells (Fig. 4b) but when restimulated with anti-CD3, less interaction of Nck with TSAd was observed (Fig. 4b and c). Similarly, TSAd promoted the interaction of Nck with Lck in JTAg cells. However less Lck was associated with Nck in anti-CD3 stimulated cells (Fig. 4d and e).

Finally we examined the influence of TSAd on Nck-Lck association in primary T cells, using CD4+ murine *SH2D2A*^*−/+*^ or *SH2D2A*^*−/−*^ blast T cells. The *SH2D2A* gene encodes TSAd. In line with the increased Lck co-immunoprecipitation with Nck in 293T and JTAg cells, more Nck associated Lck molecules was observed in *SH2D2A*^*−/+*^ blast T cells compared to the TSAd deficient *SH2D2A*^*−/−*^ blast T cells (Fig. 4f and g). Taken together, these data suggest that TSAd promotes interaction between Nck and Lck. In the presence of all three molecules, Nck may become phosphorylated by Lck.

### TSAd promotes Nck-SLP-76 interaction

**Fig. 5:**
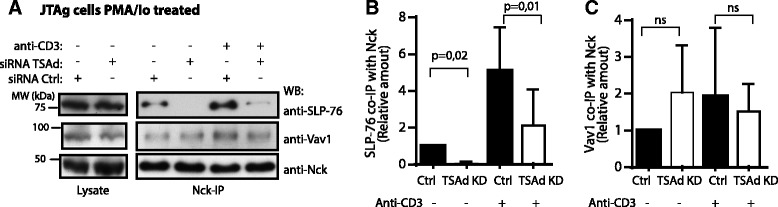
TSAd affects Nck/SLP-76 interaction. Immunoblot of Nck-IP and lysates from JTAg cells with TSAd expression (siRNA Control) or JTAg cells lacking TSAd (siRNA TSAd), as in Fig. 4b. **a** Nck-IPs of the same cells shown in Fig. 4b were immunoblotted with the indicated antibodies. Western blots are from one representative out of four (unstimulated) or three (anti-CD3 stimulated) independent experiments. **b** Densitometry analysis of the relative amounts of SLP-76 levels co-immunoprecipitated with anti-Nck antibody from (a) was performed by ImageJ analysis. The amount of co-immunoprecipitated SLP-76 in unstimulated TSAd expressing control cells was set to 1.0. Data are means ± SD from three independent experiments. P-values estimated by a 2-tailed paired *t*-test. **c** Relative amounts of Vav1 precipitated with anti-Nck antibody as in (b). ns: not statistically significant

Nck is known to bind to the cytosolic adaptor SLP-76 [13] as well as the guanine nucleotide exchange factor Vav1 [11]. Since we had established TSAd as an Nck interaction partner, and since TSAd influenced the interaction of Nck with Lck, we explored whether TSAd also influences interaction of SLP-76 or Vav1 with Nck. Western blotting of Nck immunoprecipitated from PMA/ionomycin treated JTAg cells, showed a significant reduction in Nck and SLP-76 interaction in the absence of TSAd, both in resting and CD3 restimulated cells (Fig. 5a and b). In contrast, similar amount of Vav1 was co-immunoprecipitated with Nck in the presence or absence of TSAd (Fig. 5a and c). These data suggests that TSAd not only promotes Nck interaction with Lck, but also influences Nck’s interaction with some but not all of its partners.

### TSAd promotes F-actin polymerization in T cells

**Fig. 6:**
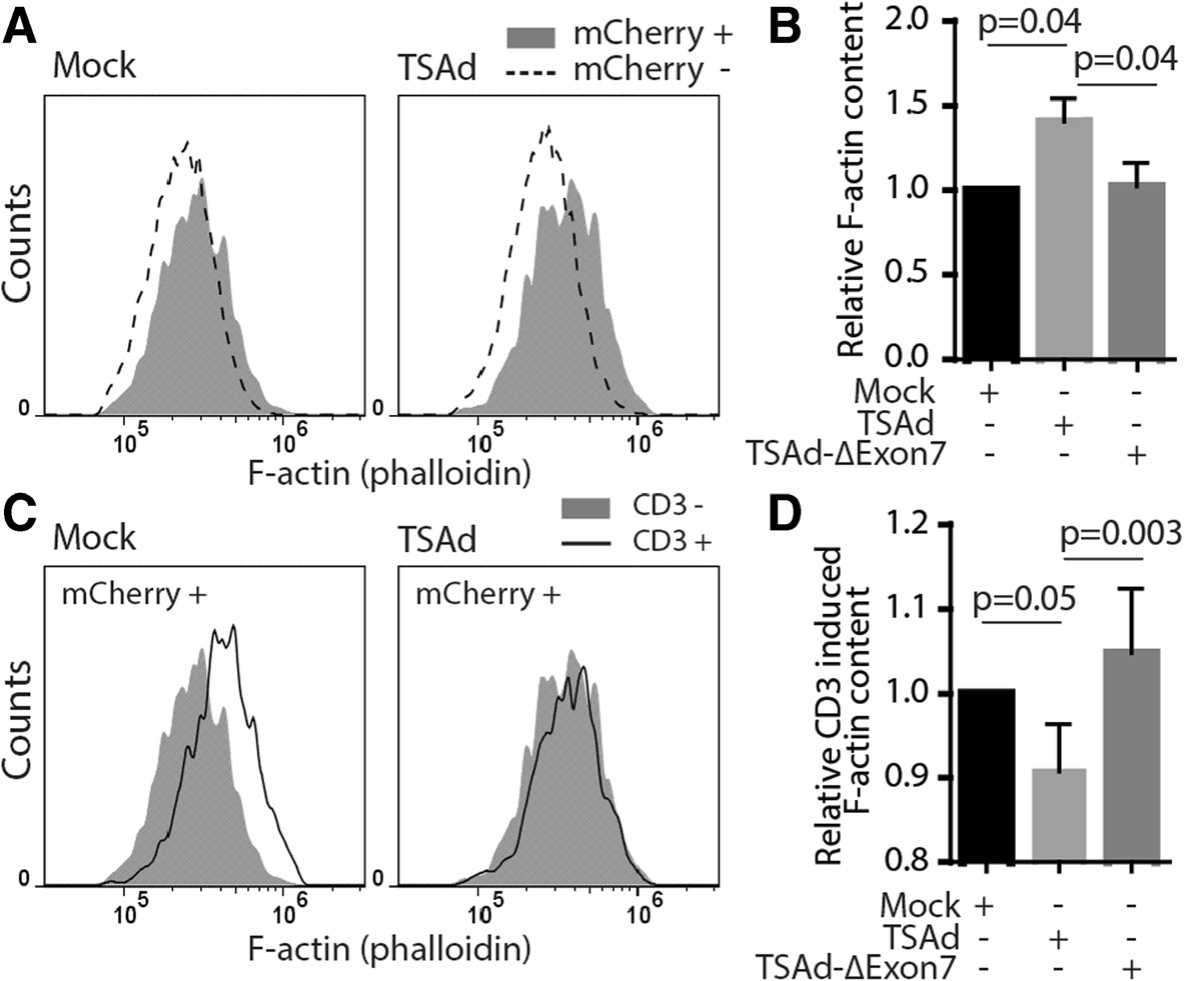
TSAd promotes F-actin polymerization in T cells. Actin polymerization in TSAd-mCherry transfected JTAg cells was quantified with imaging flow cytometer. **a** Histograms of F-actin intensity (phalloidin) in mCherry+ popoulation versus mCherry- population transfected with mCherry empty vector (mock, left histogram) and TSAd WT-mCherry (right histogram). One representative of five experiments is shown. **b** Graph shows relative amount of F-actin in resting cells shown in (a). Mean ± SD (n = 5) and p-values from 2-tailed paired *t*-test. **c** Overlay of histograms of F-actin intensity in non-stimulated and anti-CD3 (OKT3) stimulated cells for mock and TSAd WT transfected cells. Results shown are one representative of five independent experiments. **d** Graph shows relative change in F-actin content in anti-CD3 stimulated cells. Relative change of F-actin content in anti-CD3 stimulated mock transfected cells was set to 1. Data shown are means ± SD of three independent experiments. P-values estimated by a 2-tailed paired *t*-test

Nck is a known regulator of the actin cytoskeleton [9, 15, 17]. The interaction between TSAd and Nck thus led us to examine the effect of TSAd on polymerized actin (F-actin) in JTAg cells. JTAg cells were transfected with cDNA constructs encoding TSAd WT-mCherry or TSAd-mCherry lacking both the Nck SH2 and the Nck SH3 interaction sites (TSAd ∆Exon7), and amount of F-actin was quantified with IFC (Fig. 6). As an internal reference in each sample, we calculated the relative increase in F-actin in cells that were transfected (mCherry +) compared to non-transfected cells (mCherry-) (Fig. 6a). TSAd WT-mCherry transfected cells displayed significantly more F-actin than cells expressing either TSAd-∆Exon7-mCherry or mCherry alone (Fig. 6a and b). Following anti-CD3 stimulation, an increase in F-actin content was observed both in the mock transfected and the TSAd transfected cells (Fig. 6c). However, the relative increase in F-actin amount in cells expressing TSAd WT was significantly less pronounced than for cells expressing mCherry alone (mock) or TSAd-∆Exon7-mCherry (Fig. 6c and d). Taken together, these data show that TSAd influence F-actin content in both resting and CD3 stimulated cells, and that this effect is dependent on the Nck and Lck interaction sites on TSAd.

## Discussion

In this work we identified Nck as a novel interaction partner for TSAd. Through peptide array analysis, precipitation assays and co-localization studies, we found that Nck and TSAd interact both through the Nck SH2 domain and the Nck SH3 domains. Moreover, TSAd promoted interaction of Nck with Lck and SLP-76. Cells expressing TSAd displayed increased amount of polymerized F-actin, while TSAd lacking the Nck and Lck interaction sites, failed to do so. Interaction of TSAd with Nck may represent one pathway whereby TSAd modulates actin polymerization events.

We used scoring matrix-assisted ligand identification (or SMALI), a web-based program for predicting binding partners for SH2-containing proteins [20] to identify potential novel interaction partners for TSAd. The SH2 domain binding motives were identified by SMALI based on data from an oriented peptide array library (OPAL) screening. OPAL allows systematically testing the residues in the phosphopeptide one by one. The SMALI analysis covers 76 of the 120 SH2 domains in the genome. Nck1 SH2 binding to TSAd pTyr^280^ displayed the best SMALI score for that particular position in TSAd. A limitation of using OPAL screening or phage display techniques to identify SH2 domain binding motifs is that amino acids that are prohibitive for SH2 domain interaction in a given position may go undetected [25]. Also, the SMALI analysis only covers 60 % of the existing SH2 domain in the human genome. Thus it is possible that there are other SH2 domain containing proteins that also may be predicted to interact with TSAd pTyr^280^ with a similar probability.

Nck was found to interact with both TSAd pTyr^280^ and pTyr^305^, with pTyr^280^ as the preferred binding site. We have previously shown that TSAd pTyr^280^, pTyr^290^ and pTyr^305^ provide interactions sites for the Lck SH2 domain [5, 21]. Moreover, we previously showed that Lck interacts with the same TSAd PRR [18] as Nck (this paper). It thus appears that Nck and Lck may compete for the same binding sites on TSAd. However, since TSAd promoted the association of Nck with Lck, it is possible that these two molecules may also bind to TSAd simultaneously. Our current and previous data suggest that Nck- and Lck-SH2 domains have different preferences regarding the TSAd phosphotyrosines, with the pTyr^290^ [21] or pTyr^305^as the preferred Lck SH2 binding sites. Of note is that the three C-terminal tyrosines of TSAd are highly conserved [5]. This points to a critical role for these tyrosines in TSAd’s function. Similar to Lck, Nck has multiple binding sites on TSAd. This may increase the avidity between Nck and TSAd, and allow for simultaneous interaction of TSAd with both Nck and Lck. We did not directly examine whether Nck and Lck may dock onto the three TSAd pTyr simultaneously. However, one intriguing possibility is that one important function of the C-terminal TSAd tyrosines is to serve as docking sites for both the Nck and Lck SH2 domains, bringing Lck into the vicinity of Nck and its binding partners.

In pull-down assays from 293T cells, the Nck SH3 domains 1 and 3 were found to interact, presumably in a cooperative manner with the PRR of TSAd, while in Jurkat cells none of the single SH3 domains were able to pull down TSAd. This could be due to competition of individual Nck SH3 domains with other molecules present in Jurkat cells but not 293T cells, where Lck itself is a good candidate. The Nck SH3.2 domain did not show interaction with TSAd. This is in agreement with previously reported Nck SH3 interactions. Different Nck interaction partners show a clear preference to individual SH3 domains, and the interaction between Nck ligands and the different Nck SH3 domains are often found to be cooperative [10].

TSAd is expressed only at low levels in naïve T cells, thus to examine the effect of TSAd we here either used overexpression of exogenous TSAd or stimulated T cells with PMA/ionmycin or anti-CD3 to ensure expression of TSAd. In these cells, Nck and TSAd were found to constitutively interact without additional stimulation.

Both confocal microscopy and IFC supported the notion that Nck and TSAd are co-localized in the cytoplasm of unstimulated Jurkat cells. The intracellular localization of TSAd has previously been assessed [3, 18, 26]. Although both we [18] and others [26] previously noted that TSAd was also located in the nucleus, we observe mainly cytoplasmic localization of TSAd in unstimulated cells. We thus favor the notion that TSAd is primarily a cytosolic adaptor, linking Lck with some of its substrates, including also Nck.

In the presence of both TSAd and Lck, Nck phosphorylation was increased. Phosphorylation of Nck has also been reported previously. However, the physiological significance of this phosphorylation event is not clear. Nck phosphorylation is increased following TCR engagement in Jurkat cells [27]. c-Abl has been found to phosphorylate Tyr^105^ on Nck1, providing a negative feedback loop on p38 activation upon VEGF-A signaling [28]. Furthermore, Fyn has been reported to phosphorylate Nck. A model for VEGFR-2 signaling was thus proposed, whereby the phosphorylated VEGFR-2 recruits Nck via its SH2 domain, leading to phosphorylation of Nck and subsequent recruitment of Fyn and phosphorylation of Nck by Fyn [29]. We have previously reported that TSAd is recruited to the activated VEGFR-2 receptor and that in its absence, VEGF-A stimulation fails to induce actin stress fiber formation [7]. Moreover, TSAd interacts with Src in endothelial cells, and is required for activation of Src upon VEGF signaling [6]. Although it needs to be formally demonstrated, our current study suggests that TSAd may also recruit Nck to the activated VEGFR-2, providing a possible link between VEGFR-2 signaling and the actin cytoskeleton.

The role of Nck in T cell activation has been strongly debated, and reports have been contradictory. Elimination of Nck with siRNA knock down in Jurkat cells leads to cells that are hyporesponsive to TCR stimulation [30]. Recruitment of Nck via its SH3.1 domain to a PRR in the CD3ε, has been proposed to be the earliest event in TCR triggering [15, 31–34]. Still, the role of CD3ε-Nck interaction in T cell activation remains controversial [32, 35, 36]. It is possible that Nck recruits a priming tyrosine kinase to CD3ε, most likely the Src kinase Lck to allow for the phosphorylation of adjacent CD3ζ subunits [37]. It has previously been reported that Nck binds to the unique domain of Lck via its SH3 domains, and that phosphorylation of Ser^59^ disrupts the interaction [23]. Our current work suggests that TSAd may link Nck with Lck, providing a scaffold to which both molecules may bind simultaneously. Alternatively TSAd may alter Nck or Lck or both thus promoting subsequent interaction of these molecules with each other. We observed a TSAd independent interaction of Lck with Nck both in murine T cell blasts lacking TSAd, and in TSAd suppressed JTAg cells. Since we failed to observe any interaction between Lck and Nck-SH3.1-3 nor Nck-SH2 domains in pull-downs in 293T cells, a possible explanation is that a different adapter molecule exist in T cells that bridges Lck and Nck.

Whether TSAd may be a missing link between CD3ε, Nck and a kinase, is unclear. It has previously been reported that TSAd is essential for activation of Lck [38]. In contrast we have previously shown that in the absence of TSAd, CD3ζ is more highly phosphorylated upon TCR stimulation [21], and vice versa, that when TSAd is overexpressed, CD3ζ is less phosphorylated [39]. Our interpretation of this apparent paradox is that TSAd both serves to activate Lck by interacting with the Lck SH3 and SH2 domains, and at the same time provides tyrosines that are substrates for Lck [5]. TSAd may thus serve as a competitive substrate “sink” allowing temporary and spatial control of Lck activity, allowing particular substrates, such as Nck bound to TSAd, to become phosphorylated, while other substrates may be protected from Lck. With respect to the possible role of Nck-TSAd-Lck interaction for initiation of TCR signaling, one caveat is also that TSAd is expressed only at low levels in naïve resting cells. We thus favor a model where TSAd promoted interaction between Nck and Lck play a role preferentially in experienced human T cells, where TSAd expression is already induced. In experienced cells TSAd may contribute to actin dependent processes, such as migration [8, 40] or stabilization of the immunological synapse [21].

TSAd also promoted the interaction of Nck with SLP-76. Nck interacts via its SH2 domain with SLP-76 pTyr^113^ and pTyr^128^ upon TCR engagement [11, 13, 17]. Accordingly, we also observed more Nck associated with SLP-76 in our restimulated JTAg cells. In comparison Nck-Vav1 dimers are proline dependent and are constitutively formed [11, 12] which fits with our observation that the interaction between Nck and Vav1 remained unaffected by TSAd.

How TSAd may affect SLP-76 interaction with Nck remains to be studied. An intriguing observation was that upon CD3 stimulation, less TSAd and more SLP-76 was associated with Nck. Our observation that Lck is recruited to Nck in the presence of TSAd, indicates that the Lck and TSAd together primes Nck for interaction with SLP-76. Additionally, the kinase Itk is also a binding partner for both SLP-76 [12] and TSAd [8, 21, 41]. One possibility is that TSAd, Lck, Itk, Nck and SLP-76 participate in a multimolecular complex, where each of the molecules may interact with several of the other molecules in a cooperative manner.

## Conclusions

We report a novel association between TSAd and Nck. In the presence of TSAd, association of Nck with Lck as well as SLP-76 is increased. This may allow recruitment of Lck into the vicinity of Nck binding partners, and thus provide a mechanism whereby TSAd influence the actin cytoskeleton.

## Methods

### Plasmids and constructs

TSAd cDNA was cloned into the pEF-HA expression vector, and constructs encoding full-length TSAd [39], TSAd ∆239-274, TSAd ∆Exon 7 (aa 239–334) [18], single, double and triple TSAd tyrosine (Y) to phenylalanine (F) mutants [5] were cloned as previously described. Point mutations in TSAd cDNA were generated by QuickChange™ Site-Directed Mutagenesis (Stratagene). mCherry tagged TSAd constructs were cloned into the pcDNA3 vector. Nck1-GFP and GST-Nck1 constructs were provided by L. Buday [42]. The GST-Lck SH3 and GST-Itk SH3 constructs were generated as described [4, 8, 18]. All constructs were verified by sequencing.

### Antibodies

The following monoclonal antibodies were used: anti-Lck (3A5), anti-SLP-76 (F7), and anti-GST (B12) (Santa Cruz Biotechnology,Inc.), anti-HA (HA.11, Bio Site), anti-Nck (108; BD Transduction Laboratories™), anti-human CD3ε (OKT3; American Type Culture Collection), anti-phosphotyrosine (4G10; Upstate Biotechnology), and unconjugated and DyLight 488 conjugated anti-TSAd (anti-SH2D2A) (3C7, Origene). The following polyclonal antibodies were used: anti-Nck (Millipore), anti-Vav (C-14; Santa Cruz Biotechnology,Inc.) and anti-TSAd (1715 T, provided by V. Shapiro). Horse radish peroxidase (HRP)-conjugated goat anti-mouse IgG, goat anti-mouse IgG light chain specific and goat anti-rabbit IgG (all from Jackson ImmunoResearch Laboratories), and goat-anti rabbit IgG Alexa Fluor® 647 conjugate (Molecular Probes) were used as secondary antibodies.

### Expression and purification of recombinant GST-fusion proteins

GST-fusion proteins of Nck SH2, Nck SH3, Lck SH3 and Itk SH3 domains were produced in *E.coli* BL21-Codon Plus® (Stratagene) in M9 minimal salt media at 15 °C, and purified on Glutathione 4B SepharoseTM beads (GE Healthcare), according to the manufacturer’s instructions. Protein concentration and purity were analyzed by SDS-PAGE and Commassie Brilliant Blue Staining.

### Cell cultures and transfections

Human embryonic kidney (293T) cells (American Type Culture collection) and Jurkat SV40 T antigen (JTAg) cells [43] were cultured in complete RPMI (cRPMI) medium [RPMI 1640 supplemented with 5 % (293T cells) or 10 % FBS (JTAg cells), 1 mM sodium pyruvate, 10 mM Hepes, 1 % MEM non-essential amino acids, 100 units/ml penicillin, 100 μg/ml streptomycin (all from GIBCOBRL®, Life Technologies™) and 0.5 μM 2-mercaptoetanol (Sigma). Transfections of 1.5 · 10^7^ JTAg cells in RPMI 1640 with 0.5-10 μg of plasmid DNA were performed using a BTX electroporator (Genetronix) at 240 V and 25 ms. Transient transfectants were cultured for 16–24 h. 293T cells (2.2 · 10^6^) were plated in 5 ml complete media on 10 cm cell culture plates, and allowed to adhere for 24 h. The transfection solution containing 0.04-6 μg DNA and 42.4 μg/ml polyethyleneimine (PolyScience) in PBS was added drop-wise to the 293T cells. Transfected cells were further propagated for 16–24 h.

### siRNA-mediated knock down of TSAd expression

JTAg cells (1.5 · 10^7^) were transiently transfected with 1 μM solution of TSAd siRNA [7] to inhibit TSAd expression or control siRNA (24 h in total), as previously described [18]. After transfection, cells were stimulated with 50 ng/ml phorbol 12-myristate 13-acetate (PMA) and 500 ng/ml Ionomycin (Io) (both from Sigma) for 20 h to induce TSAd expression.

### Mice and propagation of murine CD4+ T blast cells

Mice used in this study were bred under conventional conditions, and approved by The National Animal Research Authority, via their local representative at the University of Oslo. The mice were regularly screened for common pathogens and housed in compliance with guidelines set by the Experimental Animal Board under the Ministry of Agriculture of Norway. TSAd deficient (*SH2D2A*^*−/−*^ or RIPB-KO*)* C57BL/6 mice were kindly provided by Professor J. A. Bluestone [41] and the *SH2D2A*^*−/−*^ mice were backcrossed >10 generations into C57BL/6 mice and maintained on a C57BL/6 background as described [7]. Murine splenocytes and lymph node cells were obtained by crushing the organs through a cell strainer (70 μm nylon, BD Biosciences). CD4+ T cells were purified from splenocytes or lymph node by negative selection (Dynal® Mouse CD4 Negative Isolation Kit (114.15), Life Technologies). The recovered population was more than 93 % CD4+ T cells, as analyzed by flow cytometry (FACS Calibur, BD Biosciences). For propagation of blast cells from primary murine CD4+ T cells, 30U mouse IL-2/ml and Dynabeads® Mouse T-Activator CD3/CD28(Life Technologies) (following the manufacturer’s protocol) was added to the cRPMI medium.

### Isolation and activation of human CD3+ T cells

Peripheral blood mononuclear cells (PBMCs) from healthy donors were isolated from buffy coats using Lymphoprep™ as previously described [44]. CD4+ T cells were isolated by negative selection (Dynabeads® Untouched Human T cells (113.44D), Life Technologies). The recovered population was more than 95 % CD3+ T cells, as analyzed by flow cytometry (FACS Calibur, BD Biosciences). Cells were activated with Dynabeads® Human T-Activator CD3/CD28 (one bead per cell), following the manufacturer’s protocol (Life Technologies) in cRPMI medium for 48 h.

### Cell stimulation and lysis

Prior to use in experiments, the anti-CD3/CD28 beads were removed from long term activated murine CD4+ T cells and cells were rested for 24–48 h before harvesting. For short time activation of JTAg cells, the cells were washed with PBS, resuspended to 5 · 10^7^ cells/ml and stimulated with 5 μg/ml anti-CD3 (OKT3) antibody for the indicated time points. In some experiments pervanadate treatment for 5 min (0,01 % H_2_O_2_, 100 μM Na_3_VO_4_) was used. Stimulation was terminated by adding ice cold PBS and centrifugation. Cells were lysed in lysis buffer containing; 1 % Nonidet-P40, 50 mM n-octyl-β-D-glucoside, 25 mM Tris (pH 7.5), 100 mM NaCl, 20 mM NaF, 1 mM Na_3_VO_4_ and 1× protease inhibitor cocktail (Roche) for 30 min on ice. For co-immunoprecipitation, reduced amounts of detergents were used; 0.25 % Nonidet-P40 and 12,5 mM n-octyl-β-D-glucoside. LDS lysis were used for immunoprecipitation in murine CD4+ T cells (final concentration) 0.1 % LDS, 1 % Triton X-100, 0.1 M LiCl, 1 mM PMSF, 5 mM EDTA, 50 mM Hepes, 1 mM Na_3_VO_4_ and 1× protease inhibitor cocktail.

### Immunoprecipitation and pull-down assays

For immunoprecipitation (IP), lysates from 293T and JTAg cells were pre-cleared three times for 30–45 min with Dynabeads Protein G (Life Technologies), and incubated with the relevant antibodies conjugated to Dynabeads Protein G (Life Technologies) for 1 h at 4 °C. For pull-down assays, cell lysates from 2.2 · 10^6^ -1.5 · 10^7^ cells were pre-cleared three times for 30 min with a 2:1 mixture of GST/Glutathione Sepharose^TM^ 4B beads (GE Healthcare), and added to aliquots of Glutathione Sepharose^TM^ 4 Fast Flow (GE Healthcare) coupled GST-fusion proteins. The mixture was rotated for 1 h at 4 °C. Beads from IP or pull-down were washed three times in 1× lysis buffer, and precipitated proteins were separated by SDS-PAGE and detected by immunoblotting. Quantification was performed on ECL autoradiography films using ImageJ software [45].

### SDS-PAGE and Western blot

Proteins were denatured in SDS loading buffer, separated by SDS-PAGE, and transferred to a PVDF membrane (Bio-Rad Laboratories) using a Hoefer Semi-Phor Semi-Dry Transfer Unit (Amersham Biosciences), or a Trans-Blot® Turbo™ Transfer System (BioRad). Blots were blocked in PBS-T (pH 7.4, 0.1 % Tween) with 3 % BSA (Biotest) or 3 % skimmed milk (Sigma), and subsequently incubated with the indicated antibodies in the appropriate blocking buffer. Signals were detected by horseradish peroxidase (HRP)-labeled secondary antibodies and Super Signal® west Pico Stable Peroxide Solution (Pierce), and developed on Amersham Hyperfilm™ ECL (GE Healthcare).

### SMALI predictions of SH2 interactions

The web based scoring matrix-assisted ligand identification (SMALI) algorithm [19, 20], was used to predict SH2 domains interacting with phosphotyrosine peptides of TSAd [NP_003966.2]. A domain scan was carried out. A relative SMALI score greater than 1.0 indicated a strong potential binding.

### Peptide spot array analysis

Peptide arrays were synthesized on nitrocellulose membranes using a MultiPep automated peptide synthesizer (INTAVIS Bioanalytical Instruments AG) as described [46]. Membranes were spotted with relevant TSAd peptides and probed with GST-tagged Nck SH2 and SH3 fusion proteins (50–150 μg/ml), followed by anti-GST antibody and anti-mouse HRP secondary antibody. Signals were detected by chemiluminescent detection by Super Signal® West Pico stable peroxide solution (Pierce).

### Confocal microscopy

JTAg transfectants were harvested 16–24 h post transfection for confocal microscopy analysis. For human CD3+ T cells activated with anti-CD3/CD28 Dynabeads, beads were removed. 1 · 10^6^ cells were washed in PBS, adhered to polylysine microscope slides (VWR) for 10 min, fixed with 4 % paraformaldehyde (Fluka Chemika) for 10 min, and rinsed twice with washing buffer (PBS, 2 % FCS). Subsequently, the cells were permeabilized with 0.1-0.3 % Triton X-100 in PBS for 5 min, stained with 2 μg/ml Hoechst 33342 (Molecular Probes) for 20 min, rinsed twice with washing buffer. Human CD3+ T cells were further stained with 5 μg/ml anti-Nck (Millipore) and anti-TSAd DyLight 488 (2.5 μg/ml) for 1 h, rinced three times and stained with goat-anti rabbit IgG Alexa Fluor® 647 (10 μg/ml) for 1 h, rinced three times with washing buffer. Both Jurkat cells and human CD3+ T cells were rinced once with dH_2_O before mounted with SlowFade® Gold (Life Technologies) and sealed with nail polish. Confocal images were acquired on an Olympus Fluoview FV1000 BX61WI upright microscope equipped with a PlanApo 100× NA 1.40 oil objective.

### Imaging flow cytometry

JTAg cells co-transfected with Nck-GFP and TSAd-mCherry constructs were harvested 16–24 h post transfection. Cells were incubated with 2 μg/ml Hoechst 33342 (Molecular Probes) for 20 min, washed in PBS, fixed with 4 % paraformaldehyde (Fluka Chemika) for 10 min, and rinsed twice with PBS. Data of 1-3 · 10^4^ cells per sample were acquired on a 12-channel ISX Imaging Flow Cytometer with 40× objective (Amnis Corporation). Samples were acquired with a bright-field (BF) area lower limit of 50 μm^2^ to eliminate debris. Single stained controls were collected with no illumination and with excitation lasers switched on. The data was analyzed using IDEAS 4.0 software (Amnis). The IDEAS compensation wizard was used to create a compensation matrix for single stained image files. The matrix was used to correct for spectral overlap in raw sample files.

### F-actin polymerization analysis

JTAg cells transfected with empty vector mCherry (mock), TSAd-mCherry or mutated TSAd-mCherry constructs were harvested 20 h post transfection. The cells were incubated with 2 μg/ml Höechst 33342 in RPMI for 20 min for nuclear staining prior to harvesting. 1 · 10^6^ cells were washed once in PBS and subsequently resuspended in 100 ul PBS and stimulated with anti-CD3 (OKT3, 10 μg/ml) for 30 s at 37 °C or PBS as unstimulated control. Stimulation of cells was terminated by addition of 100 μl fixation/permeabilization/staining solution consisting of 8 % paraformaldehyde, 0.25 mg/ml L-α-lysophosphatidylcholin) and 1,25U Alexa Fluor® 647 phalloidin (Life Technologies). Cells were washed twice with 2 % FBS in PBS (pH 7.4), and resuspended in 50 μl PBS for analysis of phalloidin intensity on a 12-channel IS Imaging Flow Cytometer with 40× objective (Amnis Coporation). The data was analysed using IDEAS 6.1 software (Amnis) and FlowJo.

### Statistical analysis

Quantitative data are shown as mean ± standard deviation. Statistical significance was defined as P- values < 0.05 and was estimated by a 2-tailed paired Student’s *t* test.

## Abbreviations

TSAd: T cell specific adaptor protein
Nck: Non-catalytic region of tyrosine kinase adaptor protein
PRR: Proline rich region
SH2: Src homology 2 domain
Lck: Lymphocyte specific protein tyrosine kinase
Itk: Interleukin 2 inducible protein tyrosine kinase
SLP-76: SH2 domain containing leukocyte protein of 76 kDa
LAT: Linker of activated T cells
APC: Antigen presenting cell
VEGF: Vascular endothelial growth factor
TCR: T cell receptor
IS: Immunological synapse
GST: Glutathione-S-transferase
FBS: Fetal bovine serum
PMA: Phorbol 12-myristate 13-acetate
IO: Ionomycin
